# Evidence of Chaos in Electroencephalogram Signatures of Human Performance: A Systematic Review

**DOI:** 10.3390/brainsci13050813

**Published:** 2023-05-17

**Authors:** Shaida Kargarnovin, Christopher Hernandez, Farzad V. Farahani, Waldemar Karwowski

**Affiliations:** 1 Computational Neuroergonomics Laboratory, Department of Industrial Engineering and Management Systems, University of Central Florida, Orlando, FL 32816, USA; c.hernandez@knights.ucf.edu (C.H.); ffaraha2@jhu.edu (F.V.F.); wkar@ucf.edu (W.K.); 2 Department of Biostatistics, Johns Hopkins University, Baltimore, MD 21218, USA

**Keywords:** chaos theory, EEG, neuroscience, nonlinear dynamical systems, performance

## Abstract

(1) Background: Chaos, a feature of nonlinear dynamical systems, is well suited for exploring biological time series, such as heart rates, respiratory records, and particularly electroencephalograms. The primary purpose of this article is to review recent studies using chaos theory and nonlinear dynamical methods to analyze human performance in different brain processes. (2) Methods: Several studies have examined chaos theory and related analytical tools for describing brain dynamics. The present study provides an in-depth analysis of the computational methods that have been proposed to uncover brain dynamics. (3) Results: The evidence from 55 articles suggests that cognitive function is more frequently assessed than other brain functions in studies using chaos theory. The most frequently used techniques for analyzing chaos include the correlation dimension and fractal analysis. Approximate, Kolmogorov and sample entropy account for the largest proportion of entropy algorithms in the reviewed studies. (4) Conclusions: This review provides insights into the notion of the brain as a chaotic system and the successful use of nonlinear methods in neuroscience studies. Additional studies of brain dynamics would aid in improving our understanding of human cognitive performance.

## 1. Introduction

The human brain is composed of 86 billion associated neurons with almost 150 trillion synapses; its structure permits the passage of electrical or chemical signals between neurons [1,2]. Brains are dynamic systems with multiple levels of nonlinearity [3]. Understanding brain functioning requires a thorough understanding of its nonlinear dynamics [3]. Communication in the neuronal network creates voltage input that can be monitored and measured by EEG, which monitors the electrical activity produced by brain cells. EEG signals derived from the central nervous system provide abundant information for neuroscience studies and reveal the neuronal network’s state [4]. Among all analytical methods to study neural networks, EEG systems are the most advantageous because they are noninvasive, user-friendly, convenient, portable, and easy to maneuver. They also achieve high temporal resolution and therefore are suitable for capturing rapid changes in neuronal states [5]. These properties make EEG an excellent tool for neuroergonomics and neuroscience studies [6].

The premise of modeling the human brain as a complex system is aimed at improving understanding of the fundamental basis of behavioral, cognitive, and perceptual processes [7]. Previously, features or descriptors of EEG signals have been captured by linear methods in the frequency domain (e.g., fast Fourier transform or wavelet transform) and parametric techniques (e.g., autoregressive modeling) [8]. Although linear techniques have yielded useful results, they have limited ability to detect the underlying nonlinear processes of EEG because the ultra-high-dimensional nature of EEG causes the signal to appear as a stochastic structure [9]. More recently, EEG signals have been found to exhibit apparent random fluctuations in amplitude over time, leading scientists to recognize EEG as a signal that displays nonlinear, dynamic characteristics. [10]. Thus, in addressing the intrinsic nonlinear nature of EEG, nonlinear dynamics theories offer a more robust approach than traditional linear EEG analysis methods [10].

Nonlinear dynamical methods provide a means for understanding the underlying brain processes in EEG signals and assessing their physiological connotations. One feature of nonlinear dynamical systems is “chaos”, which is well suited for the exploration of biological time series, such as heart rates, respiratory records, and particularly EEG [10,11,12]. The theory of nonlinear dynamical systems and chaos theory address deterministic systems that display complex and seemingly random behaviors [9]. Some studies of dynamic systems have also generated mathematical equations to predict the future states of the system by plotting its behavior trajectories [13].

In this review, we limited our search to EEG studies using chaos theory to analyze large-scale synchronization between interactive brain regions in healthy participants. The main objective of this article was to review recent studies using EEG data to analyze diverse cognitive processes by applying chaos theory and nonlinear dynamical methods. We aimed to determine whether the recognition of different brain functions and their characteristics by the application of chaos theory might contribute to a better understanding of the dynamic mechanisms underlying the brain’s cognitive performance, thus providing a complementary method to linear and traditional techniques.

This article is organized as follows. The “Methods” section presents the search strategy and eligibility criteria used to collect the articles assessed in the literature review. The “Results” section provides the literature search results, the study characteristics, a general summary of the selected articles, and answers to RQ1 and RQ2. The “Discussion” section describes the theoretical implications and applications of chaos theory in the mental processes performed in the brain and provides detailed answers to RQ1, RQ2, and RQ3. Finally, the “Conclusion” section discusses future directions, outlines challenging issues, and provides future perspectives for neuroscience in the progressing field of nonlinear dynamics.

## 2. Materials and Methods

### 2.1. Review Standards

The present literature review was conducted in accordance with the Preferred Reporting Items for Systematic Reviews and Meta-analyses (PRISMA) guidelines [14]. These guidelines outline how to combine the results of studies to perform a robust systematic review. Searches for relevant articles were based on predetermined research questions, and the specified search strategy is explained below.

### 2.2. Research Questions

- RQ1: What mental processes have been studied by nonlinear EEG analysis?
- RQ2: What are the major chaotic measures used to characterize various brain functions?
- RQ3: For non-medical purposes, what are the findings of EEG studies that have used chaos theory measures for analysis?

### 2.3. Search Strategy

Peer-reviewed research articles were extracted from PubMed, Engineering Village, Web of Science, Science Direct, IEEE Xplore, EBSCOhost, ProQuest, and Google Scholar. Articles in peer-reviewed journals and conference articles were the primary source of reviewed information. Boolean operators were applied with combinations of the following keywords in the title, keywords, or abstract: (chaos OR “nonlinear dynamics” OR “nonlinear dynamical”) AND (EEG OR electroencephalography OR electroencephalogram) AND performance. The keyword “performance” was included because we aimed to identify studies that investigated performance in aspects of the human brain using chaos measures. The date range for the search was set from 1 January 2000, to 30 April 2023.

### 2.4. Inclusion and Exclusion Criteria

To narrow the scope of the search results, we chose articles for the literature review on the basis of language, text availability, article type, and publication date. To limit the number of studies, predefined exclusion and inclusion criteria were applied. The following set criteria were used to screen identified sources: (1) studies in English only; (2) studies in peer-reviewed journals or conference publications; (3) studies performing experiments on humans; (4) studies performing experiments on healthy participants; and (5) studies using EEG as the data collection method.

EEG reflects the dynamic oscillations of the brain. Therefore, EEG analysis can detect various brain disorders in early stages, such as epilepsy, autism, depression, or dementia [13,15]. The wide range of disorders that are detectable by nonlinear methods in EEG analysis is beyond the scope of this review. Furthermore, the focus of this article was on non-medical applications of nonlinear dynamical tools; thus, the selection of articles was limited to studies in only healthy participants.

### 2.5. Data Collection and Summary of Measures

Pertinent information was extracted, as summarized in Table 1, which displays the numbers of chaos metrics, the domains of the studies, the EEG channels, the numbers of participants, experiments, and major findings.

### 2.6. Data Extraction and Synthesis

The selected articles were classified into the following six categories: (1) cognitive functions; (2) sleep, anesthesia, and fatigue; (3) emotion; (4) motor imagery (MI); (5) motor movement; and (6) resting state.

## 3. Results

### 3.1. Literature Search 

**Figure 1:**
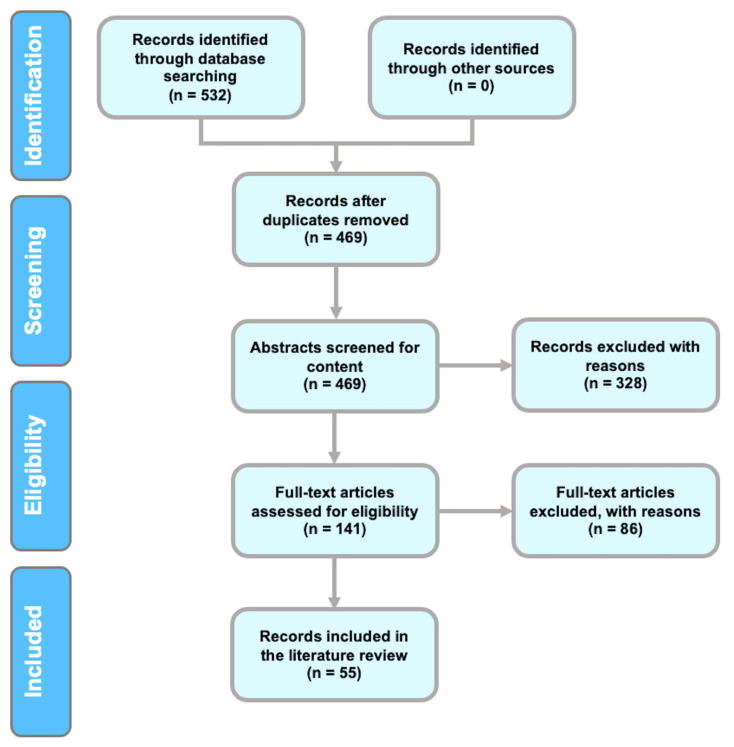
Flow diagram of the methods and selection processes used in this review, according to the PRISMA guidelines [14].

This review followed the PRISMA guidelines [14]. In Figure 1, an outline of the identification, screening, and selection of studies for inclusion in this review is presented. The first stage of the process resulted in the identification of 532 articles. After the removal of duplicates, 469 articles remained. A formal abstract screening process incorporating predefined inclusion and exclusion criteria was used to select relevant scientific articles from the remaining 469 articles. For inclusion at this stage, we used predefined inclusion and exclusion criteria. This step resulted in 141 articles being considered eligible for inclusion. A detailed examination of the full texts of these 141 articles was conducted during the selection step to ensure that they were compliant with the third step of the inclusion criteria. Ultimately, 55 publications met the criteria to be reviewed.

The sections below provide answers to RQ1 and RQ2.

### 3.2. Chaos Theory: Applications for EEG Analysis of Mental Processes

The sample size across studies ranged from 1 to 110 participants. The mean, mode, median, and standard deviation for the participants in all study samples were 24.85, 32, 17, and 25.27, respectively. 

**Figure 2:**
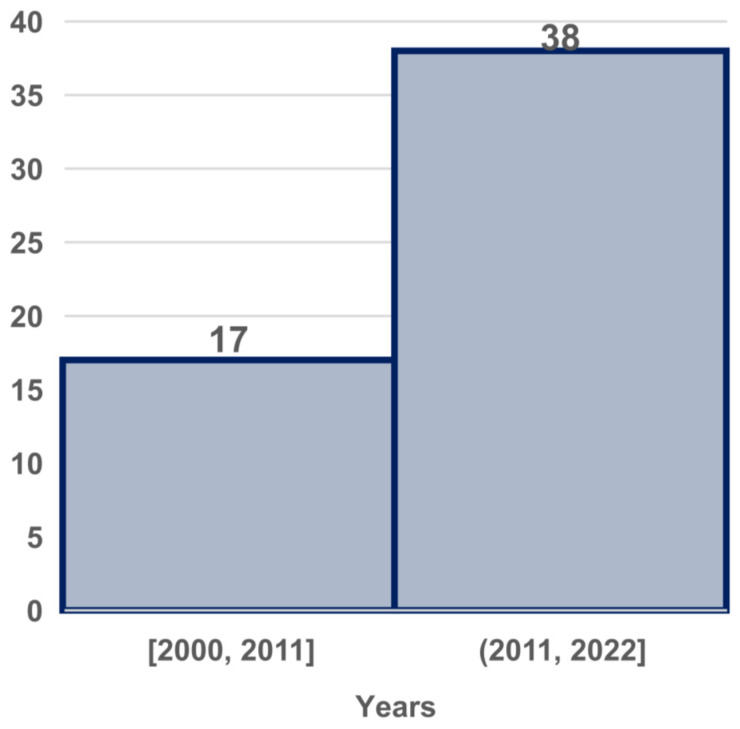
Representation of studies before and after 2011.

**Figure 3:**
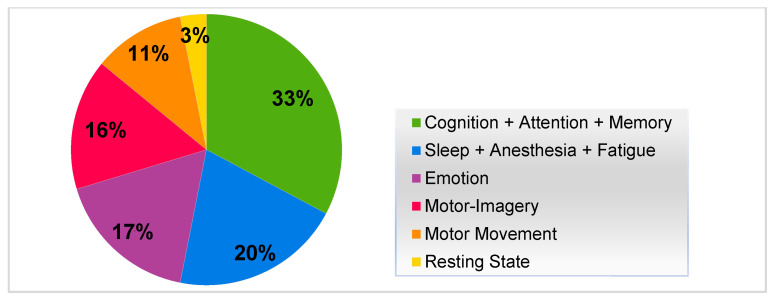
Categorization of the included studies.

The included studies were published from 2000 to 2023; Figure 2 depicts a representation of studies in the past decade and earlier, including 17 that were conducted before 2011 and 38 conducted after 2011. These studies were organized into six categories (Figure 3). A total of 33% of the studies focused on cognitive functions (cognition, attention, and memory); 20% focused on sleep, anesthesia, and fatigue; 17% focused on emotion, 16% focused on MI; 11% focused on motor movement; and 3% focused on resting state. Overall, cognitive function (32%) was more frequently addressed than other brain functions. Thus, these categories correspond to the mental processes that we sought to investigate in RQ1. Each category is explored in more depth in the discussion section.

### 3.3. Chaotic Measures of Various Brain Functions

**Figure 4:**
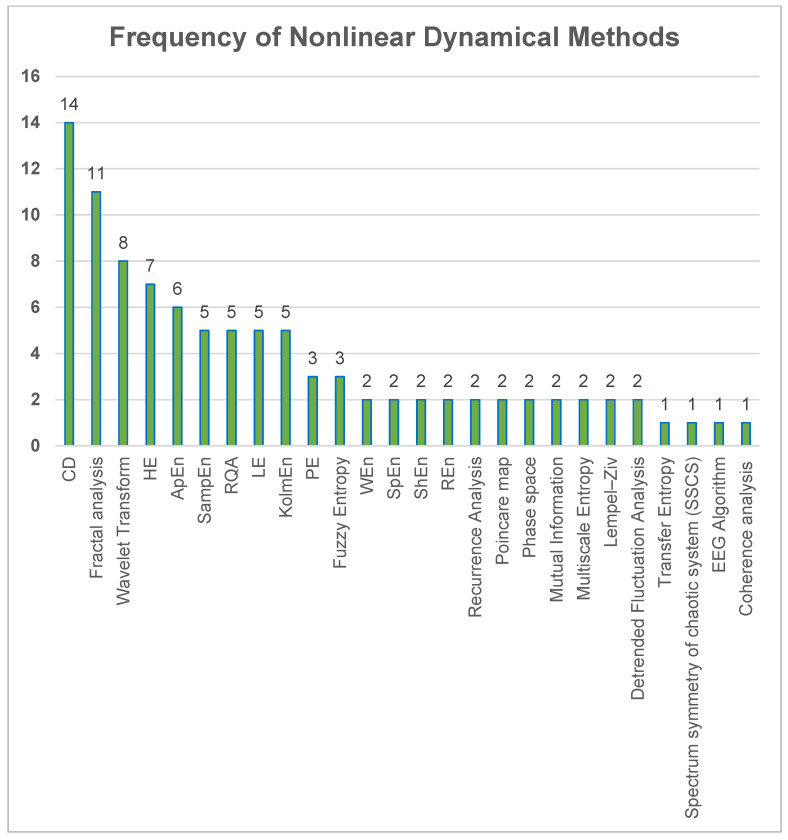
Frequency of methods utilized to analyze chaotic signals.

In all studies included in this analysis, the CD was the most frequently used nonlinear method to assess chaos (Figure 4). It was found that CD and fractal analysis techniques made the most significant contributions to chaos analysis. The major chaotic measures used to characterize different brain functions (RQ2) are shown in Figure 4. These methods are chaotic measures used to analyze EEG signals.

**Figure 5:**
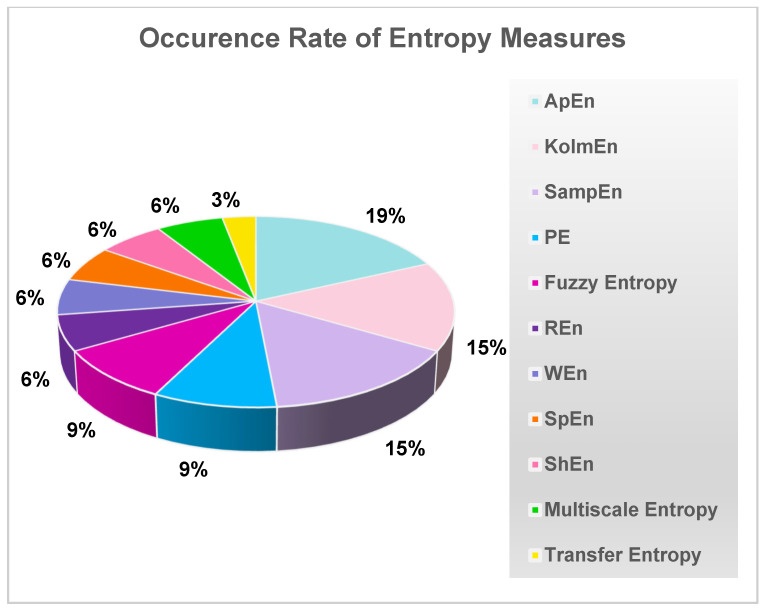
Frequency of the entropy measures among the included studies.

In studies that employed entropy metrics to examine chaos, ApEn, KolmEn, and SampEn algorithms accounted for the largest proportion of entropy methods used in these studies (Figure 5). 

**Figure 6:**
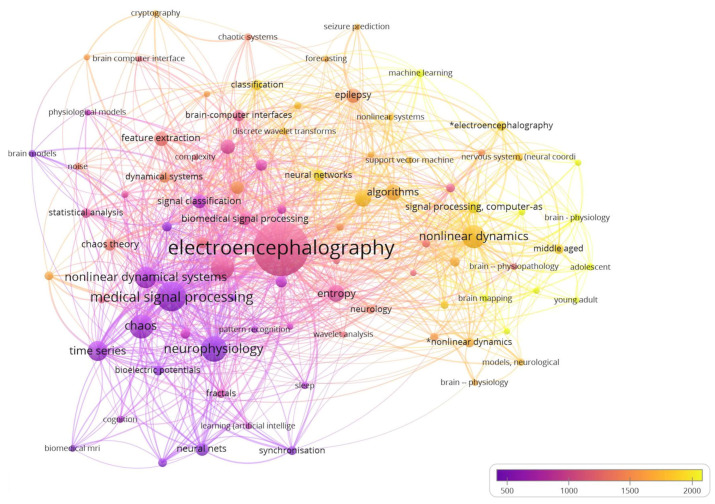
Network of keywords co-occurrence. (*) A consolidated representation of different variations of that keyword.

For the co-occurrence of keyword analysis, we set a minimum threshold of five occurrences for a keyword. Out of 1180 keywords, only 95 met this threshold and were used to create a network visualization graph using VOSviewer software (Version 1.6.19) (Figure 6). General keywords, such as human, male, female, adult, and diseases, were excluded from the analysis. The graph shows that the keywords related to electroencephalography, nonlinear dynamics, and algorithms had the highest occurrence frequency and were closely related to other relevant keywords. The strength of the links between keywords was represented by a numerical value calculated by counting the number of publications in which two keywords appear together.

## 4. Discussion

This section discusses the findings of EEG studies using chaos theory measures for analysis (RQ3). There are eight main domains: cognition, MI, motor movement, anesthesia/sleep, memory, emotion, attention, and resting state. Few studies have investigated two or more aspects of human brain functioning; hence, some overlap among studies was observed.

### 4.1. Nonlinear Dynamical Studies on Cognitive Functions

The cognitive functions of the brain refer to mental processes that enable us to receive, choose, store, modify, create, decide, and retrieve information obtained from external sources. These functions help us to better comprehend and interact with the world around us [71]. Cognitive functions are essentially brain-based abilities that are essential for performing any task, from the easiest to the most difficult. They involve the processes by which we acquire knowledge, recall information, solve problems, and pay attention [72]. As a result, this section delves into the application of chaos theory techniques in exploring these cognitive functions.

#### 4.1.1. Cognition

Cognitive processes cannot be directly measured; therefore, studies of cognitive processes usually consider some measurable activity in the brain or behavior during cognitive tasks that can connect them to cognitive processes in the brain [73].

Use of a computational approach can provide insights into how an individual neuron’s behavior and specific functions are transferred to networks and how these networks are finally merged to form complex behaviors. By analysis of nonlinear EEG signals, Ramanand et al. investigated the dynamic nature of the brain associated with mental states [21]. In their experiment, quantification of complexity by chaotic measures served as an indicator to identify active brain regions (prefrontal, frontal-temporal, central-parietal, or occipital) in specific conditions/tasks. SampEn was lower during an arithmetic task in prefrontal and occipital areas than the recorded baseline relaxed state, thereby suggesting decreased chaotic behavior of neurons in these regions while solving mathematical problems, as well as the involvement of visual areas in remembering numbers and creating a mental image of a mathematical question in the mind. The onset of the fatigue factor via physical exertion and measuring of EEG signals in participants performing the same mental calculations resulted in a diminished SampEn in the central, temporal, and parietal regions associated with performing motor actions. This evidence suggests that the onset of fatigue persists in the system by subtly changing the dynamics of the brain in the motor cortex areas and consequently influencing cognitive ability.

Synchronous oscillations in the brain are a major process for organizing and transmitting information. Local cortical activity is reflected in changes in different spectral bands and scalp locations. An increase in theta band power has been documented when working memory is loaded [28]. With attention tasks, the alpha band power decreases, thereby causing local circuits to receive more demands, particularly those in the lower alpha band and those explicitly recalling learned information in the upper alpha band [74,75]. Micheloyannis et al. reported EEG results of an arithmetic task. The CD values for all right hemisphere sites were higher, thus indicating that the right hemisphere was more active than the left hemisphere [20]. According to the study, the CD method confirmed that KolmoEn accounted for the largest proportion of entropy algorithms.

Extensive experience in applying nonlinear methods to different signals and comparisons with surrogate data to demonstrate validity have indicated that the right side of the brain exhibits more prominent engagement during arithmetic tasks. This finding would have been overlooked and missed by the spectral analysis method. Stankova et al. found that the degree of CD and intelligence level are positively correlated [17], and they concluded that the magnitude of CD is a measure of intelligence. Recorded EEG data suggested that participants with highly oscillating EEG have higher intelligence test scores than those with more periodic oscillations. This finding indicates that more intelligent individuals have a more complex and chaotic EEG structure.

The amount of order in the chaotic EEG signal is an indicator of the organization of cerebral resources in different situations [16]. The rate of chaos arising from functional processing in the brain fluctuates according to the difficulty of the cognitive task/problem. In alpha bands, the HE has a lower observed value, thus indicating greater unpredictability. The higher the delta frequencies associated with cognitive mental processing, the higher the HE values, thus suggesting that fundamental processes attract the system to more organized, self-similar, less chaotic, and more predictable conditions.

Chaos theory analysis also provides insights into EEG dynamics, which can help specialized professionals to distinguish brain activity behaviors in specialized tasks [53]. In one study, characteristics associated with individuality and professional skill were assessed with HE estimation to determine trends indicating order and chaos in time series obtained from participants’ EEG recordings. The participants engaged in tasks related to their professions while also learning a skill through consistent and repeated practice for the purpose of specialization and memory consolidation. Consequently, an ordered trend or “fingerprint” in the brains of those professionals was detected. In a sample population of pilots, the most notable functional brain characteristic was consistently higher HE in the beta band than that in another specialized population (dancers).

In comparison, dancers’ most prominent functional brain characteristic was higher HE in gamma frequencies. According to consensus, some elements of gamma-band oscillation (30–64 Hz) relate to states of attention of a spiritual nature [76,77], involving an absence of the sense of location and time. In contrast, beta-band oscillations (13–30 Hz) refer to everyday activities that require active attention and many types of cognitive demands.

One or more aspects of behavior and brain activity are usually used to study cognitive processes. In many studies, response times (RTs) have been measured as a behavioral characteristic of participants’ cognitive task performance. In the context of RTs with fractal or chaotic characteristics, determining whether brain activity (which has direct connections with cognition) can also exhibit fractal and chaotic features is relevant [78,79]. Popivanov et al. [19] sought to determine whether different output variables associated with cognition during the performance of a cognitive task might be dynamically similar. The nonlinear properties of EEG and RTs were studied for nonlinear characteristics. RTs are considered integral measures of cognitive processes in the brain, although in contrast to EEG, they do not directly represent brain activity. In that study, the participants visualized a series of MI tasks, and analysis of their RTs and EEG records revealed a clear self-similarity and chaotic behavior pattern, thus providing an understanding of how the nervous system is a self-organizing mechanism. The authors suggested that MI and phrase understanding are distinct types of cognitive processes characterized by dynamic cooperation.

Computer technology and the internet have changed how people learn and think. Currently, the use of the internet and its adverse effects on people’s behavior pose major challenges, particularly for people with internet addiction [80]. Nonlinear dynamical methods can also address the cognitive consequences of internet addiction. According to the CD and KolmEn, the EEG signals of students with internet addiction show chaotic characteristics [81]. Internet addiction in students has been associated with impaired prefrontal cortex function and a diminished ability to make decisions. Shan et al. investigated the pathological abnormalities in the prefrontal cortices of students with internet addiction to help people recover from internet addiction more effectively [18]. Analysis of the results of the chaotic features of internet-addicted students and control groups indicated that, compared with typical students, those with internet addiction have higher neuronal activity and chaotic behavior of EEG signals in the prefrontal cortex at rest. These results have led to the identification of effective treatment strategies, including counseling, and various methods of replacing interests, blocking, incentivization, and developing new habits.

#### 4.1.2. Attention

In event-associated potential studies, the oddball paradigm is a standard task for assessing cognitive ability and attention [82]. Participants perform an “oddball” task and respond to target stimuli that occur infrequently and irregularly among a series of standard stimuli [83]. Muller et al. used EEG to assess age-associated differences in nonlinear dynamical features of the brain during rest and oddball auditory performance [23]. With their eyes closed, participants listened to two types of tones: a constant 1000-Hz tone as the standard stimulus and an intermittent 800-Hz tone as the deviant stimulus. The experiment was conducted under two experimental conditions: passive listening (unsupervised) and active counting (supervised). The first condition did not require responses, whereas the second condition required the participants to count the number of deviant tones during the trial and provide feedback at the end. During the eyes closed condition, nonlinear coupling decreased with age, whereas CD increased. However, when focusing on deviant stimuli, CD (thus complexity) decreased with age, and nonlinear coupling increased.

The findings of a study by Ke et al. indicated that SampEn did very well among the dynamical parameters (ApEn, SampEn, and multi-scale entropy), with accuracies of 76.19% and 85.24% in recognizing the three levels of attention for the two experiments, respectively. SampEn also outperformed the theta/beta power ratio. These results suggest that nonlinear dynamical parameters may be essential for developing a reliable system for attention recognition [24].

Another study used a new method to measure how long people can focus their attention. The study found that people can focus their attention for longer periods of time when they receive feedback, but the level of their attention is lower in this case. The authors discovered that these results are evidence that the brain has limited cognitive resources and that these results could be useful for the development of brain-computer interfaces that can control human mental processes [25].

The continuous performance task (CPT) algorithm-based task, also known as “Sustained Attention Dots”, was the experimental task in Azarnoosh et al.’s study. Participants were asked to respond in a certain way where there was a 4-dot pattern in which there were a total of 600 patterns and 4 trials. The authors found that, using nonlinear analysis of reaction time, as well as EEG signals from the frontal and central lobes of the brain, it was possible to distinguish between attention and the onset of mental fatigue during trials. Furthermore, the changes in entropy over time demonstrated a decrease in the complexity of mental activity as fatigue set in [26].

#### 4.1.3. Memory

Continuous measurement of working memory load via noninvasive methods in participants performing a cognitive task would aid in evaluating cognitive function, thus helping people to maintain productivity and efficiency in task completion and prevent cognitive overload [84]. A nonlinear analysis of mental arithmetic tasks with varying difficulty levels performed by Zarjam et al. revealed that the frontal and occipital lobes are the regions most affected by the cognitive load of questions asked in the experiment [29]. When the questions become more challenging and frequent, the activated areas in these regions expand. The complexity in the brain increases with increasing task load, in agreement with findings from previous studies showing that the dimensionality of the brain dynamic decreases in states in which there is no overt task or the individual is in a resting state [85,86]. The decrease in HE suggests that the random behavior of the brain signals vanishes, and a more structured oscillation forms in response to the difficulty of the arithmetic task. These findings were also observed in a study by Stam [28], which evaluated a hypothesis that would help to explain the controversies among scientists regarding conclusions derived from the CD and thus the complexity of EEG data in different brain functions. Stam discussed differences among previous researchers’ findings; instead of testing the CD on the broad bandpass filter of EEG (0.5–30 Hz), he subdivided the EEG data into separate theta and lower and upper alpha bands. Working memory conditions increase the lower alpha band’s dimension. A linear analysis revealed the desynchronization of the lower alpha band. In women, compared with men, more desynchronization occurred in the theta band and the lower alpha band, and a higher CD in the theta frequency was observed. The chaotic nature of signals makes them resistant to synchronization. A correlation between working memory capacity and a smaller theta band dimension in women was observed during the no-task condition. Lower band alpha complexity can be understood as increased resynchronization associated with attention functions. The study concluded that the higher chaotic behavior and desynchronization in women than in men are closely tied to task performance.

Most studies have adopted the same approach in evaluating activity in the brain relating to memory, by selecting one or several baselines and comparing them to tasks that involve a higher level of cognitive demand or working memory demands. Ramanand et al. observed that the brain is divided into four regions: prefrontal, frontal-temporal, central-parietal, and occipital [21]. In their experiment, quantification of complexity by chaotic measures served as an indicator to identify the brain regions involved in specific conditions/tasks. The authors found that the SampEn is lower during the arithmetic task in the prefrontal and occipital regions than the during the recorded baseline relaxed state, thus suggesting decreased chaotic behavior of neurons in these regions during solving of a mathematical problem, as well as the involvement of visual regions in remembering numbers and creating a mental image of a mathematical question in the mind. When participants performed mental calculations after physical exertion, and their EEG signals were measured, there was a decrease in SampEn in the central, temporal, and parietal regions, which are involved in motor actions. This evidence suggests that the onset of fatigue persists in the system by subtly changing the dynamics of the brain in the motor cortex areas. Behzadfar et al. concluded that cortical activity during short-term memory tasks can be used to test the effectiveness of linear and nonlinear methods for feature detection [27]. The authors found that the Pen increases significantly in the frontal and occipital lobes and therefore would improve the effectiveness of memory-based neurological systems in assessing feedback.

Limitations: One of the limitations of studying cognitive processes is the inability to directly measure them. Consequently, researchers often rely on observable brain activity or behavior during cognitive tasks to establish connections with underlying cognitive processes. However, the analysis of brain activity using nonlinear methods can pose challenges in interpretation, with subjectivity potentially affecting the process.

In cognitive neuroscience, many studies have utilized chaos theory techniques, but they often encountered limitations due to small sample sizes, restricting the generalizability of their findings. Furthermore, a lack of standardization in the methods used to analyze EEG signals and quantify chaos makes it challenging to compare results across different studies.

Moreover, EEG signals have limited spatial resolution, complicating the precise identification of brain activity locations. Pinpointing specific brain regions involved in cognitive processes becomes challenging as a result.

Additionally, cognitive tasks are influenced by numerous extraneous variables, such as emotional state, fatigue, and motivation. Controlling these variables poses a challenge, and it can be difficult to isolate their effects on chaos measures.

### 4.2. Nonlinear Dynamical Studies on Sleep, Anesthesia, and Fatigue

The depth of anesthesia (DOA) during surgery must be monitored to prevent patients from becoming aware during the procedure. Complying with this requirement is essential to ensure proper DOA and to avoid accidental overdoses of potentially harmful drugs [87,88]. EEG is preferred as a modern method for assessing DOA over traditional methods based on subjective measurements, such as heart rate and pupil size [89]. Because EEG is nonlinear, chaotic parameters are appropriate to identify DOA [90]. Bai et al. developed an accurate and practical anesthesia monitoring index that can be used for sedation procedures. In that study, the dynamic features of an EEG signal were characterized using an improved version of LZC, defined by the number of distinct substrings and the rate at which they recurred in a given sequence; greater values indicated more complex data. Through the measurement of LZC, the diversity of the patterns in a signal was determined. These results were then compared with traditional LZC with entropy measures: PE, composite PE index, responsive entropy, and state entropy, which indicate the DOA. A combination of permutation and the Lempel–Ziv complexity test (PLZC) surpassed all other metrics in differentiating between awake and deep anesthesia and predicting the anesthetic drug’s effect. Because PLZC is based on the LZC algorithm and is a permutation procedure, it combines symbolic dynamic theory, probability theory, and nonlinear systems theory. This novel complexity metric is a nonparametric, easy-to-calculate index that does not require long data segments to be computed. Moreover, this metric is not based on an assumption that the time series are stationary. Thus, these features make PLZC an excellent method for analyzing EEG signals in real time [31]. Joo et al. investigated the changes in the complexity of EEG signals during general anesthesia induced by propofol. They used three different entropy measures, namely pattern entropy, local pattern entropy, and ApEn, to quantify the complexity of EEG signals. The authors found that local pattern entropy is the best measure to track the changes in the complexity of EEG signals during anesthesia, compared to pattern entropy and ApEn. Local pattern entropy showed a more robust correlation with the propofol concentration, indicating a gradual loss of complexity in the EEG signals as the subject moves from an awake to a sedated state. Overall, the study suggests that local pattern entropy is a better measure of EEG signal complexity during general anesthesia, and it has the potential to be used for real-time monitoring of anesthesia [34].

Liang et al. described a novel method called maximal overlap discrete wavelet transformation (MODWT), based on HE analysis and involving the effects of sevoflurane on the brain [32]. MODWT decomposes EEG signals into multiple time series at varying frequencies. Studied conditions included awake state, induction, deep anesthesia, and light anesthesia. Raw EEG data were divided into seven sub-bands; however, the frequency range of 0.5–12.5 Hz was found to have the greatest relevance. Increasing doses of many inhalation anesthetics tend to affect this band most strongly. HE of low-frequency bands can be used to estimate the precise moment of unconsciousness. A comparison between HE of low-frequency bands and HE of raw EEG revealed that both can distinguish an awake state from an anesthetized state. For HE of low-frequency bands, however, the reaction time for the transition from a wakeful to a moderately anesthetized state is shorter than that for HE of raw EEG. The EEG in anesthesia has been found to have an HE of <0.5, indicating non-persistent behavior. These results are consistent with those of sleep state analysis, in which HE indices decreased for all low-frequency bands as anesthesia deepened. Overall, the HE of low-frequency bands performed best in tracking sevoflurane concentrations because of their lower susceptibility to artifacts. Therefore, this method is viable for evaluating how anesthetic drugs affect brain activity.

In many DOA studies, chaotic feature extraction and neural network classifiers have been combined to achieve high accuracy in detecting anesthetic levels. By combining nonlinear chaotic measures and neural network classifiers, Lalitha et al. proposed a method for the automatic identification of anesthetic depth levels on the basis of EEG signals [30]. In the training and testing of neural network classifiers, one or several chaos measures were extracted from chaotic parameters, and the performance of the classifiers was measured for sensitivity, specificity, and overall accuracy. The results of the experiment showed that using the LE with Elman networks (feedback model) detected the optimum DOA with 99% accuracy.

Bolaños et al. monitored sedation-analgesia levels using phase diagrams or Poincaré plots [33]. The authors identified chaotic behavior in phase diagrams or Poincaré plots and distinguished it from true randomness. Thus, the Poincaré plot model is adequate for estimating sedation–analgesia levels. Poincaré plots are typically quantified using SD1 and SD2, obtained by fitting an ellipse. SD1 is the standard deviation of points perpendicular to the line of the ellipse’s identity, and the ellipse’s width is measured. Simultaneously, SD2 is the standard deviation calculated along the line of identity and is the length of the ellipse. The ratio of SD1 to SD2 in a band frequency of 30–110 Hz has shown promising results for determining sedation levels, owing to its high predictive probability. In Li et al.’s study, the method of composite multi-scale permutation entropy (CMSPE) was able to track minor transitions between light and deep anesthesia. The authors’ findings indicate that CMSPE is superior to the raw single-scale PE in demonstrating the effects of sevoflurane on the central nervous system [35].

Because of the loss of senses and movement during general anesthesia, experimental operation conditions are similar to those during sleep [91,92]. Previous research has consistently demonstrated that sleep and anesthesia share a biological mechanism, particularly the gamma-aminobutyric acid pathway [93,94,95]. Furthermore, common regions of the brain are activated during sleep and anesthesia. Because of the overlap between sleep and anesthesia studies, these studies are discussed together. According to Jeong et al. [15], CDs measured in sleep deprivation conditions are lower than those measured in normal sleep conditions [36]. The decrease in the dimensionality of EEGs in sleep-deprived states indicates decreased chaotic behavior. Biological systems have less complexity and diminished degrees of freedom when they are functionally compromised. A loss of dynamical brain responsiveness to external stimulation or inactivation of previously active networks may explain the observed dimensionality reduction, which is most apparent in the brain’s left central, right parietal, and right occipital areas.

Li et al. aimed to develop an accurate and efficient method for sleep stage classification using single-channel EEG signals. The proposed method used a cascaded SVM model that improved the overall average classification accuracy, and the study analyzed different nonlinear dynamics features and found that fuzzy entropy, LZC, SampEn, and multi-scale entropy contributed significantly to the improvement of accuracy. However, they required more time to be calculated than other features [37].

Sharma et al. proposed a flexible analytic wavelet transform (FAWT)-based method for detecting drowsiness using EEG signals, which achieved high accuracy, sensitivity, and specificity in distinguishing between alert and drowsy states. The developed FAWT-ELM-based system was shown to be effective, robust, and accurate, and it could be used to model a real-time drowsiness-detection system [38]. Additionally, Gao et al. aimed to develop a method for detecting fatigue in drivers using EEG signals. They collected EEG signals from subjects in alert and fatigue states during a simulated driving experiment. They then used a recurrence network (RN) to combine information from the EEG signals and fed the resulting data into a convolutional neural network (CNN) to extract features and classify the data as either alert or fatigued. The results showed that their proposed RN-CNN method was highly accurate, with average accuracy of 92.95%. They also compared their method with existing methods and found that it outperformed them, indicating that their method was effective in detecting fatigue in drivers [39]. Kalauzi et al. examined the patterns of brain activity, specifically alpha waves, in people when they were awake and drowsy. They found that the patterns of brain activity were more complex when people were drowsy compared to when they were awake. They also found that the traditional way of measuring complexity, namely FD, was not able to detect these differences. Instead, the authors used three new measures of complexity that they developed and were able to show that the differences in brain activity patterns were statistically significant [40]. ApEn and Kolmogorov complexity are useful measures of the dynamic complexity of EEG and have a strong association with mental fatigue. Complexity measures decrease as mental fatigue increases. In addition, kernel principal component analysis-hidden Markov modeling was proven to provide a potentially effective model for estimating mental fatigue [41].

Limitations: The studies that rely on EEG signals to monitor the DOA have encountered certain challenges. These challenges include the susceptibility of EEG signals to external factors, such as muscle movement or electrical interference, which can introduce artifacts in the signal and potentially lead to inaccurate measurements of the DOA. Moreover, some of these studies have limitations, such as small sample sizes or being conducted in a single center, restricting the generalizability of their findings.

It is worth noting that these studies primarily focused on monitoring DOA during anesthesia induction and maintenance, neglecting other crucial aspects of anesthesia, such as emergence from anesthesia or recovery time. Additionally, the complexity of mathematical and statistical analyses employed in these studies may pose difficulties in terms of replication or comprehension for clinicians without specialized training.

Another concern is that these studies may not adequately account for individual patient variability in response to anesthesia or other medications. As a result, their ability to accurately predict or prevent adverse events, such as awareness or overdose, may be compromised. These limitations underscore the need for further research and a more comprehensive approach to anesthesia monitoring and management.

### 4.3. Nonlinear Dynamical Studies on Emotion

With the increasing use of human-computer interfaces, accurate automatic emotion-detection algorithms based on EEG data have become commonplace. EEG data have been used in studies of emotion recognition because emotions and their associated responses are processed primarily in the brain [96,97,98,99]. Using machine learning techniques to identify individual emotional states enhances understanding of human emotions [43]. The number of occasions on which a human agent is replaced by an automatic emotion recognition algorithm is increasing, including lie detection, treatments for obsessive-compulsive disorder and attention-deficit hyperactivity disorder, and e-learning [42]. To distinguish among different affective states, classification techniques have been widely used. Studies using RQA with the arousal-valence model have achieved high-performance rates and accuracy.

Bahari et al. defined the arousal spectrum spanning from no arousal (bored) to excitement (alert), a valence spectrum ranging from negative (sad) to positive (happy), and liking (taste/favor) as the third and final factor incorporated into participants’ tastes [42]. Feature extraction and detection of emotions by RQA and their classification with a k-NN classifier achieved accuracy of 64.56%, 58.05%, and 67.42%, respectively, in the three categories of scaling—values much higher than the accuracy achieved by linear (spectral analysis) methods. Their findings suggest the reliability of chaotic measures in detecting emotions using EEG signals.

Fan et al. used RQA and logistic regression to classify human emotional states using nonlinear feature extraction [43]. The authors suggested that RQA achieves better classification performance than conventional power spectral features in EEG-based emotion recognition. The proposed method with selected RQA feature extraction showed a test accuracy of 75.7%. These findings may be used to identify the neurophysiology of human emotions.

Gao et al. proposed a novel algorithm called Multi-order detrended fluctuation analysis (MODFA), which improved the accuracy of EEG-based emotion recognition by measuring the homeostasis of prefrontal cortex neural activity. The results showed that MODFA outperformed conventional measures, such as fuzzy entropy and power spectral decomposition, with the best binary classification accuracy of 96.81%, the best ternary classification accuracy of 76.39%, and the best six-classification accuracy of 42.17%. Their findings also suggested that arousal had a far greater impact than valence on emotion recognition [44]. In the study by Guodong et al., the results of the experiment demonstrated that combining multiple features extracted from emotional EEG signals through multi-feature fusion produced better outcomes than using a single-feature extraction method. Using a long short-term memory neural network, the proposed method achieved high classification accuracy in emotion recognition. The method’s performance was better than that of other traditional artificial design feature-based methods that utilized SVM or DBM [45].

In another study, researchers analyzed the relationship between emotions and movement in the brain using various analysis methods, such as SampEn, transfer entropy, and mutual information. The SampEn analysis revealed changes in the EEG complex dynamic system, where different emotions caused changes in the SampEn of the frontal lobe. The transfer entropy analysis measured the strength of corticomuscular coupling, showing that happiness and sadness can promote the two-way transmission of information between the cerebral cortex and the muscle nerves and that the primary transmission of information occurs from the cortex to the muscle nerves. Last, mutual information analysis calculated the correlation between EEG signals, showing that different emotions could promote the exchange of information in specific brain regions, and grip strength could produce some long-distance exchange of information in the brain regions [46].

Tuncer et al. presented an automated EEG-based emotion recognition method using a novel fractal pattern feature extraction approach called fractal Firat pattern (FFP) and the tunable Q-factor wavelet transform (TQWT) signal decomposition technique. A multilevel feature generator was developed using FFP and TQWT. An improved iterative selector was utilized for feature selection. The model was tested on emotional EEG signals with 14 channels using linear discriminant (LDA), k-NN, and SVM classifiers. The proposed framework achieved 99.82% accuracy with the SVM classifier, indicating that FFP and TQWT-based feature generation can be successful in emotion recognition using EEG signals [47].

Khodabakhshi et al. found that the results obtained from the recurrence plot (RP) features were more statistically significant in distinguishing emotional ratings than those produced by the Poincaré map function. The RP-based approach was particularly successful in identifying levels of dominance, and of the 32 EEG electrodes analyzed, it was able to distinguish dominance levels in 23 electrodes, while the Poincaré map function only identified dominance levels in 5 electrodes. In addition, their study found that significant correlations were observed over a larger area of the cortex for all affective states when using nonlinear analysis, compared to the analysis of EEG power bands. Overall, these findings suggest that the RP-based approach is a more effective method for analyzing EEG data and identifying emotional states [48].

Yang et al. study aimed to design a reliable emotion classification system for affective computing that could enhance communication between humans and machines. The researchers conducted an emotional arousal experiment to induce three emotions—happiness, sadness, and fear—and measured the corresponding EEG signals of each subject. They proposed an RQA-based channel-frequency convolutional neural network (CFCNN) recognition system for distinguishing emotion states and found that it can achieve effective emotion classification with high accuracy and good stability, outperforming two traditional methods. Additionally, they observed that the performance of the gamma frequency band in classifying emotions shows a strong correlation between emotional processes and gamma frequency band activities. The findings suggest that the proposed recognition system has great potential for research in affective human–machine interaction systems and EEG signal identification in other areas [50]. Additionally, in the Chen et al. study, the authors compared two cases of using wavelet transform and entropy measures for feature extraction to improve the accuracy of emotion classification. They found that considering baseline data features improved the accuracy of classification, with nonlinear dynamic features leading to higher accuracy than wavelet-derived features. The most salient features were found to be a combination of ApEn and SampEn, with EEG gamma-band features being more important than other frequency bands [51].

Maity et al. investigated the effect of a simple acoustical drone stimulus on the human brain using EEG data. Nonlinear dynamical analysis techniques, such as empirical mode decomposition and multifractal detrended fluctuation analysis, were used to analyze the EEG data. The findings suggest that the input of drones enhances the complexity of alpha and theta waves, and this study has potential applications in cognitive music therapy [52].

Contrary to previous studies, Li et al. found that linear features outperformed the use of nonlinear features in each frequency band [49].

Limitations: While studies employing EEG for emotion recognition have yielded valuable insights, their applicability to real-life situations may be limited due to the controlled laboratory conditions in which the data have typically been collected. It is important to consider that the accuracy of automatic emotion-recognition algorithms varies depending on the specific method and dataset utilized. Another challenge is the presence of noise and artifacts within EEG data, which can have a detrimental effect on the accuracy of emotion recognition. Furthermore, certain emotion states may prove difficult to distinguish solely based on EEG data, necessitating the incorporation of additional physiological measures to enhance accuracy.

The use of automatic emotion recognition algorithms across different fields gives rise to ethical concerns, including privacy considerations and the potential for misuse. Since these algorithms rely on analyzing personal and intimate information, safeguarding individuals’ privacy becomes paramount. There is also a need to address the potential misinterpretation or misapplication of the emotion recognition outcomes, as well as the implications of such algorithms on personal autonomy and decision-making.

### 4.4. Nonlinear Dynamical Studies on Motor-Imagery

When visualizing an object, the brain’s cognitive function selects the appropriate grasp pattern for holding the object and then sends this decision to the neuromuscular pathway involved in executing the grasp. People with lesions in neuromuscular pathways, such as those with amyotrophic lateral sclerosis, spinal muscular atrophy, or stroke, are unable to move their limbs and perform daily activities. Nevertheless, they can visualize specific movements. Using a brain-computer interface (BCI), the human brain can communicate with the external environment. Hence, brain activity can be acquired from the brain and translated into commands to control external devices [100]. BCI has been extensively studied to create a direct communication link between the brain and prosthetic devices, thus allowing people with disabilities to live independently. MI is a passive modality involving capturing signals during the imagining of physical activities to achieve rehabilitation [54]. Functional imaging has revealed that, even in the presence of lesions, the motor system is activated during the imagining of motion [101]. The motor network can be activated even with neuromuscular impairment because MI is unaffected [102,103]. MI performance can be assessed with EEG-based MI BCI [104,105,106,107], which interprets commands from MI. Imagined hand movements may modulate brain signals, thus providing an opportunity to communicate simple messages with MI. Diaz et al. found that the brain modulates active tasks differently for MI mental processes on different timescales [55]. The study considered short-term (1 s) and long-term (2 min) time ranges. High HE in the short term indicated the modulation of the brain in response to the ongoing mental process and the long-range persistence in the active underlying structure. In contrast, the long-term timescales were non-persistent with similar HEs and were usually present in resting states.

Elbaz et al. aimed to improve the performance of BCIs by extracting different features from EEG signals and using various preprocessing, feature selection, and classification schemes. The maximum accuracy that they achieved was 90.7%, and the maximum mutual information was 0.76 bits, with the distance series features outperforming other state-of-the-art algorithms. The study’s findings suggest that nonlinear dynamical systems-based features can improve the accuracy and mutual information of BCI systems [56].

Brain signals recognize imagined hand movements and therefore can convey simple messages using imagery of hand motions [108]. To help patients with neuromuscular disorders, identifying the particular cortical region associated with a specific grasp pattern is imperative to implement BCI. Roy et al. compared different grasp patterns from EEGs and provided a robust algorithm to decode participants’ MI EEGs [54]. Their findings have suggested that the grasp pattern of a cylindrical or spherical object can be identified; the combination of CD and SVM achieved 80.6% accuracy in classifying the grasp pattern of the object of interest; therefore, the modality could be implemented to detect and classify other random items.

Hosni et al. investigated the use of graph-based RQA and complex network theory to analyze the nonlinear recurrence patterns in the mu and beta spectral bands of EEG signals during imaginary tasks. These graph-based features were called recurrence rate (RR), determinism (DET), the maximum length of diagonal lines (LMAX), laminarity (LAM), the maximum length of vertical lines (VMAX), and recurrence time entropy (RTE). These features were used to capture the nonlinear dynamics of the neural system and improve the classification of MI tasks in the study. The study found that the graph-based RQA features outperformed traditional linear spectral features, achieving an average accuracy of approximately 80%, compared to 74% for the linear features. The RQA features that were found to be most indicative of the complexity of the neural system’s dynamics were RR, DET, LMAX, LAM, VMAX, and RTE. DET and RTE features were found to be sensitive to MI neural responses, while LMAX, VMAX, LAM, and RR were more frequently selected across subjects and cross-validation folds. The authors concluded that the proposed nonlinear features could potentially improve MI-BCI performance by exploiting the nonlinear neural dynamics embedded in MI neural responses beyond the classical linear spectral characteristics. They also suggested that future works should validate the nonlinearity in the dataset using a surrogate procedure, conduct a proper statistical evaluation of the proposed nonlinear features, investigate other variations of graph-based nonlinear dynamics, and explore powerful feature selection and classification algorithms to extract robust discriminative patterns from high-dimensional nonlinear data [60]. Khare et al. proposed a new method for accurately classifying different mental tasks using EEG signals and a BCI. The method involves using the TQWT technique with automatically selected tuning parameters and then selecting important features from the resulting signals using a least squares SVM classifier. The proposed method achieved high accuracy of 99.78%, which is superior to other state-of-the-art techniques using the same database [61]. Maksimenko et al. found that a combination of delta and mu/alpha frequency bands in EEG signals can be used to extract features of brain activity associated with motor execution and MI in untrained individuals. They found that, during motor execution, there was event-related desynchronization in the mu/alpha-band in the temporal, central, and parietal lobes, and event-related synchronization in the delta-band was most pronounced in the frontal lobe. During MI, mu/alpha-band exhibited event-related synchronization, mainly revealed in the central and parietal lobes and significantly decreased in temporal lobes. MI was characterized by a significant change in frontal lobe delta activity, while motor execution was associated with event-related synchronization in the delta band. The researchers proposed a real-time algorithm to extract a single event associated with motor execution or MI from the background EEG. The algorithm was able to correctly recognize 19 motor execution events and 16 MI events out of 20 events each in an experimental session. Group analysis performed for 12 subjects demonstrated 92.9% motor execution events detected with a 5.5% false alarm rate and 81.6% MI events detected with a 9.1% false alarm rate [62].

In the visual cortex region of the brain, steady-state visual evoked potentials (SSVEP) reflect electrical activity generated by stimulation frequencies. EEG signals of SSVEP are weak, and detecting commands in the nonlinear, non-stationary, and noisy signals of EEG is challenging. A novel method of feature extraction based on chaos theory was introduced by Kai and colleagues [57]. Nonlinear chaos detection relies on nonlinear dynamics systems to detect weak signals on the basis of nonlinear chaos theory. SSVEP EEG is periodic and therefore can be incorporated as an external perturbation into systems with chaotic behavior. By detecting the change in the chaotic state after the addition of EEG of SSVEP to the chaotic system, the target frequency of SSVEP can be determined. Chaos theory is a prominent method for detecting signal features, and it shows good performance in data accompanied by noise. Traditional methods suppress noise, thus resulting in the loss of valuable information in EEG data; however, the novel method of spectrum symmetry of chaotic systems (based on chaos) has been applied to frequency detection of SSVEP data. Owing to its sensitivity to weak data and immunity to noise, this method is advantageous for target frequency detection in BCI-illiterate participants.

Another method for mapping human-imagined motor activities was developed by Baravalle et al., who described a two-dimensional representation entropy-complexity plane, assessed the dynamic nature of the signals, and deduced the emergent characteristics of the system [58]. Entropy and complexity measures (introduced by Rosso) are mutually complementary concepts. Stochastic resonance and coherence resonance describe an increase in order in a nonlinear dynamic system caused by a specific amount of noise. The principle of complexity used in this method, introduced by Rosso and Massoller, enables the separation of stochastic from chaotic time series [109]. In addition to measuring randomness, the method detects correlational structures. The entropy-complexity plane provides a global metric that illustrates many characteristics typically associated with the dynamical behavior of motor and envisioned movements.

Multi-electrode recording acquired at high temporal resolution with MI-based BCI generates data with high dimensionality. The analysis of multi-channel recordings can be adjusted to the individual characteristics of many participants; however, classifying large datasets remains computationally costly. Furthermore, because of the high redundancy of the raw data, the classification model is at risk of over-fitting. Thus, choosing a method for extracting task-relevant characteristics from data to simplify the presentation of the dataset is essential. An algorithm that decreases the dimensionality of features can enhance the efficacy and efficiency of a classification process in general. Some methods used for MI feature extraction include band power [110], variants of autoregressive models [111], and common spatial patterns (CSPs). Because CSPs perform relatively better in distinguishing among classes of data, it is the most widely used algorithm to enhance the classification of MI signals [112,113]. The effectiveness of REn (a general version of ShEn for feature extraction of MI movements to assist BCI technology as a feature extraction method in multi-class MI systems) has been compared with the CSP method [59] and found to achieve superior results. REn has also been compared with other chaotic-inspired feature extraction methods, including Katz and Higuchi, which were used for BCI systems in earlier studies [59]. Among the three chaotic measures, REn has the highest classification accuracy. Furthermore, the accuracy and convenience of REn make it a suitable tool for feature extraction of MI systems.

Beyond the application of chaos theory to BCI technology, another study sought to identify a potential biomarker to identify the parallel and cross-functional nature of cognitive phenomena that manifest simultaneously and over time in the brain during the execution of any challenging task [55]. The authors found recurrent patterns in how intraindividual and interindividual differences are manifested through EEG signal analysis. The findings indicated specialization in frontal, temporal, and occipital areas and interhemispheric interaction controlling the chaotic/non-chaotic balance in the brain in participants imagining a choreographed dance. The dancers were asked to use their imaginations to design the movement and choreography of a performance. They were required to address two aspects of the task: the creative component and the technical component. Regarding the first task, most individuals preferred working in a more fluid environment, wherein a daydream-like state was entered to optimize the interplay of options and creative purposes. Meanwhile, another part of the process might require more instantaneous, mid-analytical decision-making, involving more self-organization, long-term memory, and persistence. The study suggested that the brain modulates its ongoing processes on two time scales. Modulation in the EEG signals of the short time scale involves refined, instantaneous supervision, as indicated by high HE values, thereby demonstrating the presence of long-term, continuous processes in the working brain structure. In contrast, on the long time scale, the modulation has a short memory and non-persistent behavior, as well as similar values for HE to what were found recently in resting conditions for the EEG alpha band [55].

Limitations: Participants in studies involving people with disabilities may have varying degrees of impairment, which can affect the accuracy of results and the ability to generalize findings to broader populations. Although BCIs have shown promise in improving the quality of life of people with disabilities, they still have limitations in terms of accuracy and reliability. The accuracy of BCIs may be affected by factors such as the quality of the EEG signal, the type of signal processing algorithms used, and the ability of the user to consistently produce the desired brain signals.

### 4.5. Nonlinear Dynamical Studies on Motor Movement

Voluntary movements are preceded by complex brain processes, which emerge earlier in the supplementary and primary motor areas and later in other brain regions. Pre-movement neuronal activity correlates with movement planning and initiation, which are cognitive processes. Approximately 2 s after the initial movement is executed, supplementary motor and motor cortex areas exhibit activation. KolmEn has revealed that the EEG patterns in the supplementary motor, premotor, and motor areas of the brain are synchronized in a nonlinear chaotic manner, and are associated with the stages of preparation, intention, decision-making, and the initiation of voluntary movements [63]. Dushanova et al. found similar results in participants presented with a target cue. In their study, to reach the target on a screen display, participants were required to maneuver a control device as part of the voluntary movement; after reaching the target, they were required to press a switch. EEG signals analyzed with chaos metrics revealed three distinct periods of high complexity that may be interpreted as phases of movement organization. The two periods before the movement onset might indicate that the participants were modeling the movement as part of the earliest stages of preparation. MI, attentional focusing, and short-term memory updating may occur during this period. The third-period dynamics shift from a low level of complexity (when participants are ready to reach the target and press the switch) to a higher level when participants are close to reaching the target. The phases in voluntary movement organization are equivalent to these periods. The regions of high dynamic complexity are followed by areas of high predictability and low dimensionality, thus suggesting that the distant cortices operate synchronously. The incremental rise in values that indicate chaos may be attributable to successive phases of brain movement organization [64].

Continuous motor output adaptation is a function of the integration of frontal, parietal, and sensorimotor brain activity [114,115]. High-frequency synchronization among the visual, parietal, and motor cortices indicates that neural coherence or synchronization across remote brain regions may be part of the mechanism that facilitates visuomotor integration [116,117]. To determine whether an improvement in task performance affects activity within the network active during the different stages of a visuomotor integration, Kranczioch et al. examined the effects of changing network activity during various stages [66]. The authors evaluated learning-associated changes in EEG and brain activity in a visually guided, real-time feedback-based task, providing real-time tracking and modification of motor performance. A coherence analysis was used to determine high-frequency synchronization among the visual, parietal, and motor cortexes. A phase coherence analysis was performed on the signals at different regions to analyze their phase relationships. An absence of synchronization was represented by a phase coherence value of 0, whereas synchronization was indicated by a value near 1. Their results have demonstrated that motor performance learning and advancement are coupled with different coherence patterns for different stages of motor performance. Particularly in the period before movement initiation, the frontal-central and parieto-occipital regions appear to show high coherence and a concurrent decrease in coherence between frontal-central and parieto-occipital regions, thus suggesting the strengthening of underlying neural networks. When movement is initiated, the improvement in complex, continuous movement appears to be due to activity in an ipsilateral-medial network, despite initially appearing to be more dependent on the contralateral network. The sensorimotor processing load during hand gripping is placed on ipsilateral centro-parietal brain regions.

The chaos/order balance in brain processes can be detected from the observed individual differences. As the brain develops, it can be expected to be a system that learns successful correlations between order and chaos and between competition and cooperation. The human brain will operate progressively until it reaches the lowest energy consumption and the highest efficiency possible in the given situation. Depending on the infinitely varying possibilities of the paths (trajectories) followed by the brain during its learning process, infinitely different strategies are expected to be used for solving problems, requiring the same energy and information processing resources, thereby achieving a chaos/order and cooperation/competition equilibrium [55]. Hung et al. studied rifle shooting experts and amateur shooters firing 40 shots in the standard standing position while EEG data were recorded [65]. The experts achieved the tasks using fewer neural resources and exhibited a greater level of behavioral output despite a continuing cognitive challenge. An essential aspect of this performance was refining cognitive processes to the point at which the task was executed automatically. The experts’ lesser reliance on complex brain activities during target shooting explains why their performance was more precise and less variable than that of the amateur shooters. The amateur shooters exhibited more unstable and noisy brain signals owing to inadequate practice and minimal refinement of brain processes, thus leading to more variable performance. When the brain is in a simplified or refined mental state, it has fewer options for how to act, which can lead to more consistent performance. In contrast, when the brain is more complex, it has more options for how to act, which can lead to less consistent behavior. Among the experts, the CD analysis and target firing accuracy were inversely correlated such that lower EEG dimensionality was observed with high shooting performance. Among the amateurs, an opposite relationship was observed. Hence, greater complexity due to higher CD was associated with better performance by the amateurs. Research on motor skill learning has generally indicated greater involvement of structures such as the premotor and motor cortexes, somatosensory areas, and basal ganglia compared to other brain structures. Therefore, the prefrontal areas of the brain are more activated in amateurs, and subcortical processes replace the prefrontal area as skill acquisition progresses, thus substantially decreasing prefrontal involvement. Increasingly complex brain activation might assist amateurs in skill acquisition until a threshold, beyond which further increases in complexity would probably impair performance. A refined and efficient cerebral cortex translates into better performance on visuomotor tasks. Neuromotor noise in the brain may decrease interference with a targeted performance when complexity is diminished. During an experiment in a study performed by Yargholi et al., subjects performed tasks that involved ideomotor responses, hallucinations, motor challenges, memory recall, and post-hypnotic suggestion. Therefore, the experiment was designed to assess the participants’ hypnotic susceptibility and how it affected different cognitive processes leading to motor execution. This study found that certain brain regions, particularly those on the left side of the brain, were more efficient at distinguishing between hypnotizability levels. The authors found that brain wave patterns in people performing the same type of task were similar across different brain regions, suggesting that there may be common patterns of brain activity associated with specific types of tasks [67]. The results of the experiments and performance tests demonstrated that the suggested modeling approach is efficient in the context of movement-related potentials (MRPs), particularly for binary BCIs intended to aid severely disabled individuals, such as those with amyotrophic lateral sclerosis, in communicating or controlling devices.

Usakli applied nonlinear dynamics and fractal analysis to analyze MRPs in EEG signals. They used the variance FD method as a feature extraction tool and showed that the multifractal dimension technique can be used to model MRPs for BCI applications. The study found that the classification of EEG recordings for different tasks performed in their experiment can be distinguished using this method, and the output of this classification process can be used for communication/control. However, the classification performance was lower than that of the SVM method. They also discovered that the window size and the number of features of the time series in the EEG signal should be optimized to achieve effective multidimensional modeling. According to this study, the chaoticity of the EEG signals depends mainly on two factors: the window size of the signal and the physical proximity of EEG channels. They found that small time delays (<15 ms) and neighbor channels yield attractors that are not completely strange, as expected. This study also noted that the CP4 channel is the most efficient channel for feature extraction of MRPs for the tasks analyzed [68].

Limitations: Studies of motor movement have mainly focused on the neural mechanisms underlying motor planning and execution and have not provided information about other factors that may influence motor learning and performance, such as motivation, attention, and feedback. The studies mainly involved motor tasks that are simple and controlled and may not be representative of real-world motor tasks that are complex and unpredictable. Therefore, the ecological validity of the findings may be limited.

### 4.6. Nonlinear Dynamical Studies on Resting State

The brain is active even when it is not performing conscious cognitive tasks, and it frequently engages in involuntary wandering thoughts in a state known as the resting state [118]. Studies of brain dynamics have extensively used resting state analysis. The literature has indicated that microstate analysis can be used to investigate resting state brain activity [119]. According to the principles of microstate analysis, at any given time, only one microstate is active, and EEG scalp topologies move discontinuously among four quasi-stable states at peaks in the global field potential (GFP). These microstates are all mediated by visual, auditory, salience, and attention processes. Microstate analysis relies on several critical suppositions because it ignores all EEG data outside the GFP peaks and then clusters EEG scalp topology at the GFP peaks on the basis of the assumption that one microstate is active at a given time. Shaw et al. investigated this traditional view of microstate analysis with nonlinear dynamical methods to test the validity of these assumptions [69]. Higher complexity and chaotic behavior demonstrated that the microstate regions compete with one another. Therefore, the simple view of one microstate being active and the others being at rest is incorrect. The complex dynamics in the phase space, the high FD, and the positive LE does not support the “winner takes all” assumption of microstate analysis.

Surprisingly, the accuracy of classification reached 97.29% using linear features in the study by Zhao et al., whereas it was only 44.14% with nonlinear dynamic features. Based on the experiment’s results, it appears that the linear features of EEG, such as center frequency, max power, power ratio, average peak-to-peak value, and coefficients, although autoregressive, may perform better in individual identification than the nonlinear dynamic parameters of EEG [70].

Limitations: Resting state EEG studies may be confounded by factors such as medication use, sleep quality, and other physiological or environmental factors. These factors may limit the validity of the results and make it difficult to generalize the findings to other populations. Additionally, resting state EEG studies have typically measured brain activity in a limited frequency range, such as the alpha or beta range. This range may not capture the full range of brain activity, especially in the context of chaos theory analysis, which involves nonlinear dynamics across multiple frequency bands.

## 5. Conclusions

This review was aimed at introducing the notion of the brain as a chaotic system and demonstrating how nonlinear methods have successfully been used in neuroscience studies. Studies are increasingly using chaos theory, including useful analytical tools based on chaos theory for describing brain dynamics. We provided an in-depth analysis of the computational methods proposed to uncover brain dynamics for non-medical applications, and we explored the use of chaos theory in studying several aspects of the human brain that can be divided into six primary domains: (1) cognitive functions; (2) sleep, anesthesia, and fatigue; (3) emotion; (4) motor imagery (MI); (5) motor movement; and (6) resting state. Many higher-level functions of the brain fall under cognition, and many types of cognitive processes exist, including attention and memory. Because these psychological functions are performed by different lobes and regions of the brain, we devoted a separate section to each domain in this review.

In the studies covered in this review, CD and fractal analysis were the most frequently used methods for measuring chaos. In addition, in the analysis of results using entropy methods, researchers have primarily used ApEn, KolmEn, and SampEn. 

The advances in nonlinear dynamics and nonlinear time series analysis have substantially enabled EEG-based applications. Compared with conventional/linear approaches, which overlook the most valuable data, nonlinear methods provide better results because many brain experiments are complex and analyze large datasets. A better understanding of the dynamics of normal and pathological brain states and better tools for nonlinear time series analysis will be essential for the future of nonlinear EEG analysis.

Although a wide range of classic and novel measures are available for estimating the chaotic properties of the brain, they all share the step of quantifying distances between states in phase space. Consequently, time-delay embedding can be described by two parameters: lag and embedding dimension. In the analysis of nonlinear systems, the proper selection of these parameters is critical and challenging [13]. Because filtered noise time series can present a false impression of low-dimensional data, nonlinear measures impose limitations in interpretation [120]. EEG was the subject of the present review, but a more general review should address all types of brain measurements. It should be noted that one limitation of the current study was the lack of studies of neurological diseases, such as epilepsy, Alzheimer’s disease, etc., which might have provided valuable insights into the applications of chaos theory analysis in clinical settings.

Over the past few years, new and improved methods for nonlinear time series analysis have been pursued, and this progress is expected to continue in the future. New methods are being developed to extract novel features found in nonlinear dynamical systems in EEG signals. To address the gaps in EEG chaos theory analysis identified in our discussion, it is necessary to develop novel techniques that can overcome the limitations of the existing methods. These limitations include the requirement for long and high-quality recordings, as well as the difficulty of analyzing nonstationary, features of noise and the dimensionality of EEG signals. A persisting challenge is developing new techniques that can handle these data and produce reliable results. Moreover, to better interpret the results of chaos theory analysis in EEG, it is crucial to contextualize them in terms of the underlying neural processes that govern brain activity. Additionally, enhancing the analysis of psychological functions through chaos theory analysis would benefit from identifying the sources of inter-subject variability in EEG signals. By addressing these research gaps, we can advance our understanding of the brain’s complexity and improve the applicability of EEG chaos theory analysis to a range of fields. A continuing need exists for studies of brain dynamics. Understanding how brain dynamics are associated with structural and behavioral properties would particularly enable insights to guide future research directions.

## Figures and Tables

**Table 1:** Summary of selected articles, including the numbers of EEG channels, chaos theory metrics, numbers of participants, domains, experiments, and summaries of findings. Legend: Hurst exponent (HE), fractal dimension (FD), Lyapunov exponent (LE), correlation dimension (CD), Shannon wavelet entropy (ShEn), Rényi entropy (REn), spectral entropy (SpEn), Kolmogorov entropy (KolmEn), wavelet entropy (WEn), approximate entropy (ApEn), sample entropy (SampEn), permutation entropy (PE), recurrence quantification analysis (RQA), Lempel-Ziv complexity (LZC), and motor imagery (MI).

Article	Chaos Metric	Domain	Number of EEG Channels	Number of Participants	Summary of Experiments	Summary of Findings
[16]	HE	Cognition	14 EEG channels	12	Raven’s test was performed.	Observed HE had a lower value in alpha bands, thus indicating higher unpredictability. The HE values increased the delta frequencies associated with cognitive mental processing, thus suggesting that fundamental processes attract the system to more organized, self-similar, less chaotic, and more predictable conditions.
[17]	CD	Cognition	18 EEG channels	77	A domino test was performed, in which participants identified the correct piece corresponding to the previous patterns of dominos.	Intelligence levels and the degree of intelligence levels were positively correlated. Data suggested that participants with highly oscillating signals scored higher on intelligence tests than those with more periodic oscillations. Thus, intelligent participants appeared to have a more complex and chaotic EEG structure.
[18]	CD and KolmEn	Cognition	64 EEG channels	6	Resting state EEG was examined.	A comparison of the chaotic features of internet-addicted students and controls of typical students indicated that the former have higher levels of neuronal activity and higher chaotic behaviors of EEG signals in the prefrontal cortex at rest.
[19]	FD and CD	Cognition	9 EEG channels	42: male = 20, female = 22	Participants were asked to listen to 25 verb-and-object sentences and to understand and imagine the action indicated.	The response times and EEG records of participants visualizing a series of MI tasks clearly demonstrated self-similarity and chaotic behaviors, thus explaining how the nervous system is a self-organizing mechanism.
[20]	CD	Cognition	28 EEG channels	2: male = 1, female = 1	Participants were presented with stimuli on an LCD screen in the categories of arithmetic, language, and symbol processing and were asked to determine whether the stimulus was in the correct or incorrect domain.	The CD method indicated that the right side of the brain exhibited more prominent engagement during an arithmetic task. This finding would have been missed by the spectral analysis method.
[21]	SampEn	Cognition, working memory, attention	128 EEG channels	6: male = 3, female = 3	The following were examined: first, a passive state with eyes closed; second, performance of a mental task; and last, performance of the same mental task followed by a fatigue-causing physical task.	SampEn was diminished during the arithmetic task in the prefrontal and occipital regions with respect to the recorded baseline relaxed state, thus indicating less chaos. When fatigue factors were present, diminished SampEn in the central, temporal, and parietal regions associated with performing motor actions was observed. Fatigue changes the dynamics of the brain in the motor cortex areas.
[22]	LE, FD, and SampEn	Cognition (attention, memory), attention	19 EEG channels	4	EEG signals in response to four categories of cognitive tasks were investigated—focus, problem solving, memory, and emotion recognition. Cognitive load was assessed using the NASA Task Load Index sheet. The EEG session was designed with the eyes open condition, and a qualitative assessment was performed to determine the difficulty level of executing the task.	This study established a spatiotemporal descriptor for perceiving changes in the properties of the EEG signal, which occurs in response to cognitive load. The study used three different nonlinear parameters to analyze the EEG signals, LE, FD, and SampEn. The researchers used these findings to create a classification model that can identify different states of the brain based on the changes in the EEG signal.
[23]	CD and two coupling measures: pointwise trans-information and pointwise conditional coupling divergence (PCCD)	Attention	58 EEG channels	9	Participants with eyes closed listened to two types of tones: a constant 1000-Hz tone as the standard stimulus and an intermittent 800-Hz tone as the deviant stimulus. Participants were asked to count the number of deviant tones and provide feedback.	In the deviant stimulus experiment, nonlinear coupling increased with age, while CD/complexity decreased.
[24]	ApEn, SampEn, multi-scale entropy	Visual Attention	19 EEG Channels	14: male = 7, female = 0	Participants were required to maintain their gaze fixed on the center of a computer monitor. Although the time courses of the two experiments were the same, the difference between them was in the action phase: Experiment 1 displayed a cartoon character playing ball, while Experiment 2 showed a cartoon character walking.	The findings indicate that SampEn fared very well among the dynamical parameters, with accuracies of 76.19% and 85.24% in recognizing the three levels of attention for the two experiments, respectively. SampEn also outperformed theta/beta power ratio. These results suggest that nonlinear dynamical parameters may be essential for developing a reliable system for attention recognition.
[25]	EEG algorithm	Attention	5 EEG channels	12	A visual task that incorporates perception and primary processing of visual information.	The results obtained suggest that cognitive resources are finite, and to sustain optimal performance over an extended period, the brain needs to operate under a “safe-mode” regime.
[26]	Symbolic dynamics	Attention and Mental Fatigue	11 EEG channels	20: male = 20, female = 0	Continuous performance task (CPT) algorithm-based task called “Sustained Attention Dots.” Participants were asked to respond in a certain way where there was a 4-dot pattern. There were a total of 600 patterns and 4 trials.	The findings indicated that, using nonlinear analysis of reaction time, as well as EEG signals from the frontal and central lobes of the brain, it was possible to distinguish between attention and the onset of mental fatigue during trials. Furthermore, the changes in entropy over time demonstrated a decrease in the complexity of mental activity as fatigue set in.
[27]	WEn, ApEn, PE, FD	Short-term memory	2 EEG channels	16	Participants were shown 12 black and white pictures for 10 s and then asked to close their eyes for 2 min and provide the names of the pictures that they could remember.	PE increased significantly for FD, ApEn, root mean square, and waveform length in the frontal lobe and the occipital lobe, thus improving the effectiveness of memory-based neurological systems assessing feedback.
[28]	CD	Working memory	21 EEG channels	21: male = 9, female = 12	Participants recalled any of the 12 black and white pictures shown to them within 1 min of their eyes closing.	Working memory conditions increased the lower alpha band’s dimension. A correlation was observed between greater working memory capacity and a smaller theta band dimension in women during the no-task condition. Lower band alpha complexity is associated with attention functions. Higher chaotic behavior and desynchronization in women are closely associated with task performance.
[29]	CD, HE, ApEn	Working memory	32 EEG channels	1: male = 0, female = 1	An arithmetic task with seven levels of difficulty, from very low to extremely difficult, was shown on a computer display. Participants were asked to select the correct answer using a mouse button.	The decrease in the HE suggests that the random behavior of the brain signals vanishes, and a structured oscillation forms in response to the difficulty of the arithmetic task.
[30]	CD, LE, HE	Anesthesia	N/A	5	After premedication with morphine and atropine, five participants underwent anesthesia with propofol intravenously, followed by a thiopentone injection.	By combining nonlinear chaotic measures and neural network classifiers, anesthetic depth levels based on EEG signals can be identified. Using the LE with Elman networks (feedback model) detects the optimum depth of anesthesia with 99% accuracy.
[31]	Permutation Lempel-Ziv complexity (PLZC)	Anesthesia	3 EEG channels	20: male = 10, female = 10	Sevoflurane gas was administered to participants for 2 min at 3%, followed by 7% in group 1, and intravenous propofol was delivered to group 2.	A permutation Lempel-Ziv complexity test (PLZC) surpassed all other indices in differentiating between awake and deep anesthesia and in predicting the anesthetic drug’s effect.
[32]	HE	Anesthesia	3 EEG channels	16: male = 0, female = 16	During the first 2 min of sevoflurane administration, the vaporizer delivered 3% of the inspired concentration to participants, followed by 7%.	The novel method maximal overlap discrete wavelet transformation (MODWT) based on HE analysis was used to describe the effects of sevoflurane on the brain. HE of low-frequency bands (HEOLFB) tracked sevoflurane concentration best and was less susceptible to artifacts than the other methods examined.
[33]	Poincaré map (phase-space), SpEn	Anesthesia	3 EEG channels	110	Ultrasonographic endoscopy was performed under sedation-analgesia.	The Poincaré plot model is effective in estimating sedation–analgesia levels. The ratio of the ellipse’s width in the Poincaré plot to its length in a band frequency of 30–110 Hz showed promising results for determining sedation levels, owing to high prediction probability.
[34]	Lumped PE	Anesthesia	2 EEG channels	16	During general anesthesia, subjects were administered propofol continuously and its plasma concentration was measured at preset intervals. The EEG states of awake, sedative, and deep anesthesia were identified and the loss and regain of consciousness were determined through verbal commands. As the propofol infusion ended, the plasma concentration decreased gradually, leading to the subject regaining consciousness.	The study investigated the complexity of EEG signals during propofol-induced general anesthesia using three different entropy measures. The authors found that local pattern entropy (LPE) was the best measure to track changes in EEG complexity during anesthesia, showing a stronger and more robust correlation with propofol concentration. The study suggests that LPE could be used for real-time monitoring of anesthesia.
[35]	Composite multi-scale permutation entropy (CMSPE)	Anesthesia	3 EEG channels	18	EEG data were collected from patients under sevoflurane anesthesia.	Composite multi-scale permutation entropy (CMSPE) was able to track minor transitions between light and deep anesthesia. The results indicate that CMSPE is superior to the raw single-scale PE in demonstrating the effects of sevoflurane on the central nervous system.
[36]	CD	Sleep	16 EEG channels	32: male = 16, female = 16	Participants’ EEG was recorded after 8 h of nighttime sleep at 7:00 AM. Participants were then subjected to 24 h of sleep deprivation, and their EEGs were re-recorded at 7:00 AM.	CDs measured under sleep deprivation conditions were lower than those measured under normal sleep conditions. The decrease in the dimensionality of EEGs in sleep-deprived states indicated decreased chaotic behavior.
[37]	REn, LZC, multi-scale entropy, SpEn, SampEn, fuzzy entropy	Sleep	2 EEG channels	Sleep-EDF database (8 subjects)	Each recording in the database is typically 8 h long and contains information about the sleep stages of the individual. The recordings are divided into 30-s epochs, and each epoch is labeled with the corresponding sleep stage. The sleep stages are Wake, Non-REM (NREM) Stage 1, NREM Stage 2, NREM Stage 3, and REM (Rapid Eye Movement) sleep.	The study aimed to develop an accurate and efficient method for sleep stage classification using single-channel EEG signals. The proposed method used a cascaded SVM model that improved the overall average classification accuracy, and the study analyzed different nonlinear dynamics features and found that fuzzy entropy, LZC, SampEn, and multi-scale entropy contributed significantly to the improvement of accuracy. However, these factors required more time to be calculated than other features.
[38]	Flexible analytic wavelet transform (FAWT)	Sleep (Drowsiness)	32 EEG channels	16: male = 16, female = 0	Collected from MIT/BIH dataset of physiobank—no explanation of the experiment.	This study proposes a flexible analytic wavelet transform (FAWT)-based method for detecting drowsiness using EEG signals, which achieved high accuracy, sensitivity, and specificity in distinguishing between alert and drowsy states. The developed FAWT-extreme learning machine-based system was shown to be effective, robust, and accurate, and it could be used to model a real-time drowsiness detection system.
[39]	Recurrence network-based	Sleep (fatigue)	40 EEG channels	10: male = 8, female = 2	Simulated driving experiment	The study aimed to develop a method for detecting driver fatigue using EEG signals. They collected EEG signals during a simulated driving experiment and used a recurrence network and a convolutional neural network to extract features and classify the data as alert or fatigued. The results demonstrated high accuracy with an average accuracy of 92.95%, and the proposed method outperformed existing methods, indicating that it was effective in detecting driver fatigue.
[40]	FD, circular complexity, longitudinal complexity, intersecting complexity	Sleep (Drowsiness)	14 EEG channels	10: male = 7, female = 3	No task, eyes closed in a dark room	This study looked at the patterns of brain activity, specifically alpha waves, in people when they were awake and drowsy. They found that the patterns of brain activity were more complex when the person was drowsy compared to when he or she was awake, and the traditional way of measuring complexity, called FD, was not able to detect these differences. Instead, the researchers used three new measures of complexity that they developed and were able to show that the differences in brain activity patterns were statistically significant.
[41]	ApEn, Kolmogorov complexity (Kc)	Sleep (fatigue)	32 EEG channels	50	Three experimental tasks: Vigilance task—participants had to click the right mouse when an odd number appeared on the screen. Addition and subtraction arithmetic calculation of four one-digit numbers Simple switch task	ApEn and Kolmogorov complexity (Kc) are useful measures of the dynamic complexity of EEG and have a strong association with mental fatigue. Complexity measures decrease as mental fatigue increases. Additionally, KPCA (kernel principal component analysis)-HMM (hidden Markov model) was proven to be a potentially effective model for estimating mental fatigue.
[42]	RQA	Emotion	32 EEG channels	10	A total of 40 videos were presented to the participants in each trial, and self-assessment tests were performed	Feature extraction and detection of the emotion through recurrence plot analysis and k-NN classification had accuracy of 64.56%, 58.05%, and 67.42% in all three categories of scaling—values much higher than the accuracy achieved with linear (spectral analysis) methods.
[43]	RQA	Emotion	32 EEG channels	Group 1: 19; Group 2: 10	Participants scored each video that they were shown on a scale of 0 to 9 in four dimensions: arousal, valence, liking, and dominance.	With RQA, better classification performance was achieved over feature extraction works based on spectral analysis methods in emotion recognition. The proposed method with selected RQA feature extraction resulted in test accuracy of 75.7%. The findings may be used to identify the neurophysiology of human emotions.
[44]	Multi-order detrended fluctuation analysis (MODFA), fuzzy entropy	Emotion	9 EEG channels	87: male = 31, female = 56	The study involved a baseline period during which participants had their eyes open and closed while gazing at a white cross displayed on a black screen. Participants then watched six video clips with different emotional themes, including neutral, fear, sadness, happiness, anger, and disgust. The videos were used to elicit emotional responses and were entitled “World Heritage in China”, “The Conjuring”, “Nuan Chun”, “Top Funny Comedian”, “Never Talk to Strangers”, and “The Fly”.	The study proposed a novel algorithm called multi-order detrended fluctuation analysis (MODFA), which improved the accuracy of EEG-based emotion recognition by measuring the homeostasis of prefrontal cortex neural activity. The results showed that MODFA outperformed conventional measures, such as fuzzy entropy and power spectral density with the best binary classification accuracy of 96.81%, the best ternary classification accuracy of 76.39%, and the best six-classification accuracy of 42.17%. The findings also suggested that arousal had a far greater impact than valence on emotion recognition.
[45]	Fuzzy entropy	Emotion	32 EEG channels	32	EEG and peripheral physiological signals were recorded after participants watched 40 1-min music video clips.	The results of the experiment demonstrated that combining multiple features extracted from emotional EEG signals through multi-feature fusion produced better outcomes than using a single-feature extraction method. Using a long short-term memory neural network, the proposed method achieved high classification accuracy in emotion recognition. The method’s performance was better than other traditional artificial design feature-based methods that utilized SVM or DBM.
[46]	SampEn, transfer entropy, mutual information	Emotion	64 EEG channels	24: male = 10, female = 14	20 neutral, 20 happy, and 20 sad movie clips were displayed for subjects in three sessions according to the emotions portrayed. During each session, the subjects performed 15 grip strength trials and five no-grip strength trials while watching the video clips.	Researchers investigated the relationship between emotions and movement in the brain using various analysis methods, such as SampEn, transfer entropy, and mutual information. The study found that different emotions cause changes in the SampEn of the frontal lobe, and happiness and sadness can promote the transmission of information between the cerebral cortex and muscle nerves. Additionally, mutual information analysis showed that different emotions can promote the exchange of information in specific brain regions.
[47]	Fractal Firat pattern (FFP), tunable Q-factor wavelet transform (TQWT)	Emotion	14 EEG channels	28	The experiment involved four different types of games: funny, boring, horror, and calm. Each game was recorded for 5 min, resulting in a total recording time of 20 min for all four games combined.	The study proposed an automated EEG-based emotion recognition method using fractal Firat pattern (FFP) and tunable Q-factor wavelet transform (TQWT) signal decomposition technique. A multilevel feature generator was developed using FFP and TQWT, and an improved iterative selector was utilized for feature selection. The proposed framework achieved 99.82% accuracy with the SVM classifier, indicating that FFP and TQWT-based feature generation can be successful in emotion recognition using EEG signals.
[48]	Poincaré map function, recurrence plots (RPs)	Emotion	32 EEG channels	32	Participants watched 40 one-minute videos and rated each video on arousal, valence, liking, dominance, and familiarity.	The study compared the effectiveness of recurrence plot (RP) features and Poincaré map function in analyzing EEG data and identifying emotional states. The results showed that RP features were more statistically significant in distinguishing emotional ratings, particularly in identifying levels of dominance, in more EEG electrodes. The study also found that nonlinear analysis using an RP-based approach was more effective in identifying emotional states and showed significant correlations over a wider area of the cortex compared to the analysis of EEG power bands.
[49]	CD, KolmEn, ShEn	Emotion	32 EEG channels, 62 EEG channels	Group 1: 32, Group 2: 15	The experiment utilized two publicly available datasets, DEAP and SEED, to analyze EEG data from human subjects. DEAP consisted of data from 32 subjects who watched one-minute-long music video clips and rated their emotional experiences on a two-dimensional scale of arousal and valence. SEED included data from 15 subjects who watched 15 film clips to induce emotions with three classes of emotions (positive, neutral, negative) evaluated, and each class had five corresponding film clips.	The individual beta rhythm achieved the best performance, and the higher-frequency beta rhythm and gamma rhythm bands performed better than the lower-frequency theta rhythm and alpha rhythm bands. Using linear features outperformed the use of non-linear features in each frequency band.
[50]	RQA	Emotion	40 EEG channels	5	This experiment involved participants watching eight movie clips while their emotions were recorded. Each clip was preceded by guiding words displayed on the screen for 5 s, after which the clip was played, and participants were asked to concentrate on it. After each clip, participants were asked to report their true emotional state and rate the intensity of their emotions on a 10-point scale.	The study aimed to design an emotion-classification system for affective computing that could improve communication between humans and machines. The researchers induced three emotions in participants, measured corresponding EEG signals, and proposed an RQA-based channel-frequency convolutional neural network (CFCNN) recognition system for distinguishing emotion states. They found that the system achieved high accuracy and stability, outperforming traditional methods, and they observed a strong correlation between emotional processing and gamma frequency band activities, suggesting potential for research in affective human-machine interaction systems and EEG signal identification in other areas.
[51]	Wavelet transform, WEn, ApEn, SampEn	Emotion	32 EEG channels	32	Subjects watched 40 clips from music videos as emotional stimuli.	The authors compared two cases using wavelet transform and entropy measures for feature extraction to improve the accuracy of emotion classification. They found that considering baseline data features improved the accuracy of classification, with nonlinear dynamic features leading to higher accuracy than wavelet-derived features. The most salient features were found to be a combination of ApEn and SampEn, with EEG gamma-band features being more important than other frequency bands.
[52]	Wavelet transform, multifractal detrended fluctuation analysis (MFDFA), and HE	Emotion	19 EEG channels	10: male = 6, female = 4	Subjects listened to the sound of a tempura drone generated by software.	This study investigated the effect of a simple acoustical drone stimulus on the human brain using EEG data. Nonlinear dynamical analysis techniques, such as empirical mode decomposition and multifractal detrended fluctuation analysis (MFDFA), were used to analyze the EEG data. The findings suggest that the input of drones enhances the complexity of alpha and theta waves, and this study has potential applications in cognitive music therapy.
[53]	HE	MI, cognition	14 EEG channels	Group 1 (pilots): male = 10 female = 2; group 2 (dancers): male = 3 female = 7	Dancers mentally visualized and imagined a series of steps and choreographed moves. Pilots were asked to solve a short version of Ravens’ test.	When a skill is learned through repeated practice and specialization or during memory consolidation, professionals within that specialized field may share a structure of ordered trends or “fingerprints”.
[54]	CD	MI	29 EEG channels	18: male = 10, female = 8	Participants visualized the grasping of a specific object. Six objects were selected for this experiment: a can, a box, a cup, a ball, a lid, and an egg.	The grasp pattern of a cylindrical or spherical object was identified, and the combination of CD and a SVM achieved 80.6% accuracy in classifying the grasp pattern.
[55]	HE	MI	14 EEG channels	20: male = 20, female = 0	Nine professional dancers imagined performing a choreographed dance, and EEG data were recorded for 2 min	The long-term timescales in the resting state demonstrated anti-persistence and similar HEs. This finding suggests that the brain modulated its active tasks differently between the two timescales.
[56]	Wavelet transform	MI	3 EEG channels	1: male = 0, female = 1	Subjects were seated on a chair with armrests while imagining left- and right-hand motions.	The maximum accuracy achieved was 90.7%, and the maximum mutual information was 0.76 bits, with the distance series features outperforming other state-of-the-art algorithms.
[57]	Spectrum symmetry of chaotic system (SSCS), based on chaos	MI	8 EEG channels	32: male = 16, female = 16	Visual stimulation was performed.	Conventional methods for analyzing EEG data tend to filter out noise, which can result in the loss of valuable information. However, a new approach based on chaos theory, called the spectrum symmetry of a chaotic system, was used to analyze SSVEP data frequencies. This method is particularly advantageous for detecting target frequencies in BCI-illiterate participants since it is sensitive to weak signals and immune to noise
[58]	ShEn	MI	64 EEG channels	87	The experiment consists of a one-minute baseline run with eyes open and another one-minute baseline run with eyes closed. This step is followed by three 2-min runs of four different tasks. Two pairs of tasks, one run performing the physical task and the next run imagining performing the same task.	The authors were able to distinguish between motor movements and imagined movements. The authors note that the entropy measures and complexity measures are concepts that complement each other. The entropy-complexity plane provides a global metric that illustrates a variety of characteristics typically associated with the dynamical behavior of motor and envisioned movements.
[59]	REn	MI	Group 1: 3; group 2: 64; group 3: 118; group 4: 118; group 5: 22	Group 1: 1, group 2: 1, group 3: 5, group 4: 1, group 5: 9	Participants were given visual cues and stimuli for each dataset and asked to perform a specific MI task	Among the chaotic feature extraction methods used in this study, REn had the highest classification accuracy. Furthermore, the accuracy and convenience of REn make it a suitable tool for feature extraction in MI systems.
[60]	RQA	MI	13 EEG channels	6	Subjects had two sessions in which EEG data were recorded for MI tasks and periods of rest. Each session had three runs, with each run consisting of 40 trials of either MI or rest, based on visual cues on a screen. The MI task required participants to imagine moving their left or right hand, and each trial lasted for 10 s, followed by a rest period.	The study investigated the use of graph-based RQA and complex network theory to analyze the nonlinear recurrence patterns in the mu and beta spectral bands of EEG signals during MI tasks. The graph-based features outperformed traditional linear spectral features, achieving an average accuracy of approximately 80%. The study concluded that the proposed nonlinear features could potentially improve MI-based brain-computer interface performance by exploiting the nonlinear neural dynamics embedded in MI neural responses beyond the classical linear spectral characteristics.
[61]	TQWT	MI	118 EEG channels	5	Subjects performed MI tasks with their right foot (RF) and right hand (RH).	The study proposes a new method for accurately classifying different mental tasks using EEG signals and a brain-computer interface. The method involves using the TQWT technique with automatically selected tuning parameters and then selecting important features from the resulting signals using a least squares SVM classifier. The proposed method achieved high accuracy of 99.78%, which is superior to other state-of-the-art techniques using the same database.
[62]	Multifractal analysis, wavelet transform	Motor movement, MI	19 EEG channels	12: male = 9, female = 8	Participants were instructed to perform two types of tasks, lift their right hand slowly in the shoulder joint, and imagine such a movement during a given time interval. The experiment lasted approximately 30 min and was split into 10 sessions, with each session containing 20 identical events. There were five sessions of real movements followed by five sessions of imaginary movements.	Researchers have identified specific frequency bands in EEG signals that can be used to extract features of brain activity associated with motor execution and imagination in untrained individuals. During motor execution, there was a decrease in mu/alpha-band and an increase in delta-band activity in different areas of the brain. During motor imagination, there was an increase in mu/alpha-band activity and a significant decrease in delta-band activity in certain areas of the brain. The researchers developed a real-time algorithm to extract motor execution or motor imaginary events from EEG signals, which demonstrated high accuracy in detecting these events in experimental sessions with subjects.
[63]	KolmEn	Motor movement	9 EEG channels	12	Participants performed thumb movements.	Supplementary motor and motor cortex areas exhibit activation approximately 2 s after the initial movement is executed. The EEG patterns in the supplementary motor, premotor, and motor areas of the brain are synchronized in a nonlinear, chaotic manner and are associated with the stages of preparation, intention, decision-making, and the initiation of voluntary movements.
[64]	CD, KolmEn, and LE	Motor movement	9 EEG channels	19	A screen with targets was presented to the participants. To reach the target, participants maneuvered a control device and pressed a switch after reaching the target.	According to the results of this study, EEG signals analyzed with chaos metrics show three distinct periods of high complexity that can be interpreted as phases of movement organization.
[65]	CD	Motor movement	10 EEG channels	11: male = 3, female = 8	Rifle shooting experts and amateur shooters fired 40 shots in the standard standing position while EEGs were recorded.	Experts showed less reliance on complex brain activities during shooting, owing to a refinement of cognitive processes, whereas greater complexity due to higher CD was associated with better performance in amateurs.
[66]	Coherence analysis	Motor movement	64 EEG channels	11	Participants used a power handle to track a target force by applying varying forces. The participants were provided with ongoing force output feedback on a screen. The participants were required to continuously adjust their exerted force to meet the target force level.	Motor performance learning and advancement are coupled with different coherence patterns in different stages of motor performance.
[67]	RQA	Motor movement, attention, and memory	19 EEG channels	32: male = 32, female = 0	The tasks that followed involved ideomotor responses, hallucinations, motor challenges, memory recall, and post-hypnotic suggestion. Therefore, the experiment was designed to assess the participants’ hypnotic susceptibility and how it affected different cognitive processes.	This study found that certain brain regions, particularly those on the left side of the brain, were more efficient at distinguishing between hypnotizability levels. Finally, the researchers found that brain wave patterns in people performing the same type of task were similar across different brain regions, suggesting that there may be common patterns of brain activity associated with specific types of tasks.
[68]	Variance fractal dimension (VFD)	Motor movement	64 EEG channels	1: male = 1, female = 0	Three tasks: Right foot up Lip pursing Combination of the first two tasks	The results of the experiments and performance tests demonstrate that the suggested modeling approach is efficient in the context of movement-related potentials, particularly for binary brain-computer interfaces intended to aid severely disabled individuals, such as those with amyotrophic lateral sclerosis, in communicating or controlling devices.
[69]	FD, phase-space, and LE	Resting state	62 EEG channels	12	Resting state EEG was examined.	The traditional view of microstate analysis was investigated. The notions that microstate regions compete and the simple view in which one microstate is active while the others are at rest are incorrect. The complex dynamics in the phase space, the high FD, and the positive LE support this finding.
[70]	ApEn, LE, CD	Resting state	1 EEG channel	10: male = 6, female = 4	Data were collected when participants had their eyes closed and their heads still.	The accuracy of classification reached 97.29% using linear features, whereas it is only 44.14% with nonlinear dynamic features. Based on the experiment’s results, it appears that the linear features of EEG, such as center frequency, max power, power ratio, average peak-to-peak value, and coefficients of the autoregressive model, may perform better in individual identification than the nonlinear dynamic parameters of EEG.

## Data Availability

Not applicable.
